# Rejuvenation by cell reprogramming: a new horizon in gerontology

**DOI:** 10.1186/s13287-018-1075-y

**Published:** 2018-12-17

**Authors:** Rodolfo G. Goya, Marianne Lehmann, Priscila Chiavellini, Martina Canatelli-Mallat, Claudia B. Hereñú, Oscar A. Brown

**Affiliations:** 1 Institute for Biochemical Research (INIBIOLP) - Histology B & Pathology B, School of Medicine, National University of La Plata, CC 455, 1900 La Plata, Argentina; 2 Institute for Experimental Pharmacology Cordoba(IFEC), School of Chemical Sciences, National University of Cordoba, Cordoba, Argentina

**Keywords:** Aging, Epigenetics, Rejuvenation, Cell reprogramming, Therapeutic potential

## Abstract

The discovery of animal cloning and subsequent development of cell reprogramming technology were quantum leaps as they led to the achievement of rejuvenation by cell reprogramming and the emerging view that aging is a reversible epigenetic process. Here, we will first summarize the experimental achievements over the last 7 years in cell and animal rejuvenation. Then, a comparison will be made between the principles of the cumulative DNA damage theory of aging and the basic facts underlying the epigenetic model of aging, including Horvath’s epigenetic clock. The third part will apply both models to two natural processes, namely, the setting of the aging clock in the mammalian zygote and the changes in the aging clock along successive generations in mammals. The first study demonstrating that skin fibroblasts from healthy centenarians can be rejuvenated by cell reprogramming was published in 2011 and will be discussed in some detail. Other cell rejuvenation studies in old humans and rodents published afterwards will be very briefly mentioned. The only in vivo study reporting that a number of organs of old progeric mice can be rejuvenated by cyclic partial reprogramming will also be described in some detail. The cumulative DNA damage theory of aging postulates that as an animal ages, toxic reactive oxygen species generated as byproducts of the mitochondria during respiration induce a random and progressive damage in genes thus leading cells to a progressive functional decline. The epigenetic model of aging postulates that there are epigenetic marks of aging that increase with age, leading to a progressive derepression of DNA which in turn causes deregulated expression of genes that disrupt cell function. The cumulative DNA damage model of aging fails to explain the resetting of the aging clock at the time of conception as well as the continued vitality of species as millenia go by. In contrast, the epigenetic model of aging straightforwardly explains both biologic phenomena. A plausible initial application of rejuvenation in vivo would be preventing adult individuals from aging thus eliminating a major risk factor for end of life pathologies. Further, it may allow the gradual achievement of whole body rejuvenation.

## Rejuvenation: a perennial dream

The longing of man for eternal youth is universal and time immemorial. Initially sought in religions, during the Middle Ages, alchemists, a blend of mystics and proto-chemists, tried to synthesize a mysterious potion, the elixir of eternal youth, able to confer indefinite youth to those that dare to drink it. Now, it seems that science has found the biological fountain of rejuvenation—the cytoplasm of the oocyte.

The story of biological rejuvenation began in the early 1960s, with the discovery of animal cloning in frogs by John Gurdon and collaborators [1]. Mammalian cloning was achieved 30 years later, in 1996, with the birth of Dolly, the sheep [2]. Cloning of other mammalian species followed soon. It was clear that the cytoplasm of a mature oocyte contained molecules able to turn a somatic nucleus into an embryonic one that could direct the development of a new individual. At the time, it was assumed that in the oocyte’s cytoplasm, there should be a complex constellation of reprogramming factors, necessary to reprogram a somatic nucleus. However, 10 years later, Takahashi and Yamanaka [3] demonstrated that the transfer of only four master genes, namely *oct4*, *sox2*, *klf4*, and *c-myc* (OSKM genes), to adult mouse fibroblasts was able to reprogram them, taking the cells to a pluripotency stage in which they behave like embryonic stem cells. Cell reprogramming had been born, an advance that paved the way for the subsequent implementation of cell rejuvenation. The studies on rejuvenation so far published are summarized below.

## The achievement of cell and animal rejuvenation

To our knowledge, the first study reporting cell rejuvenation was published in 2011 [4]. It was a seminal piece of work that merits to be described in some detail.

**Fig. 1:**
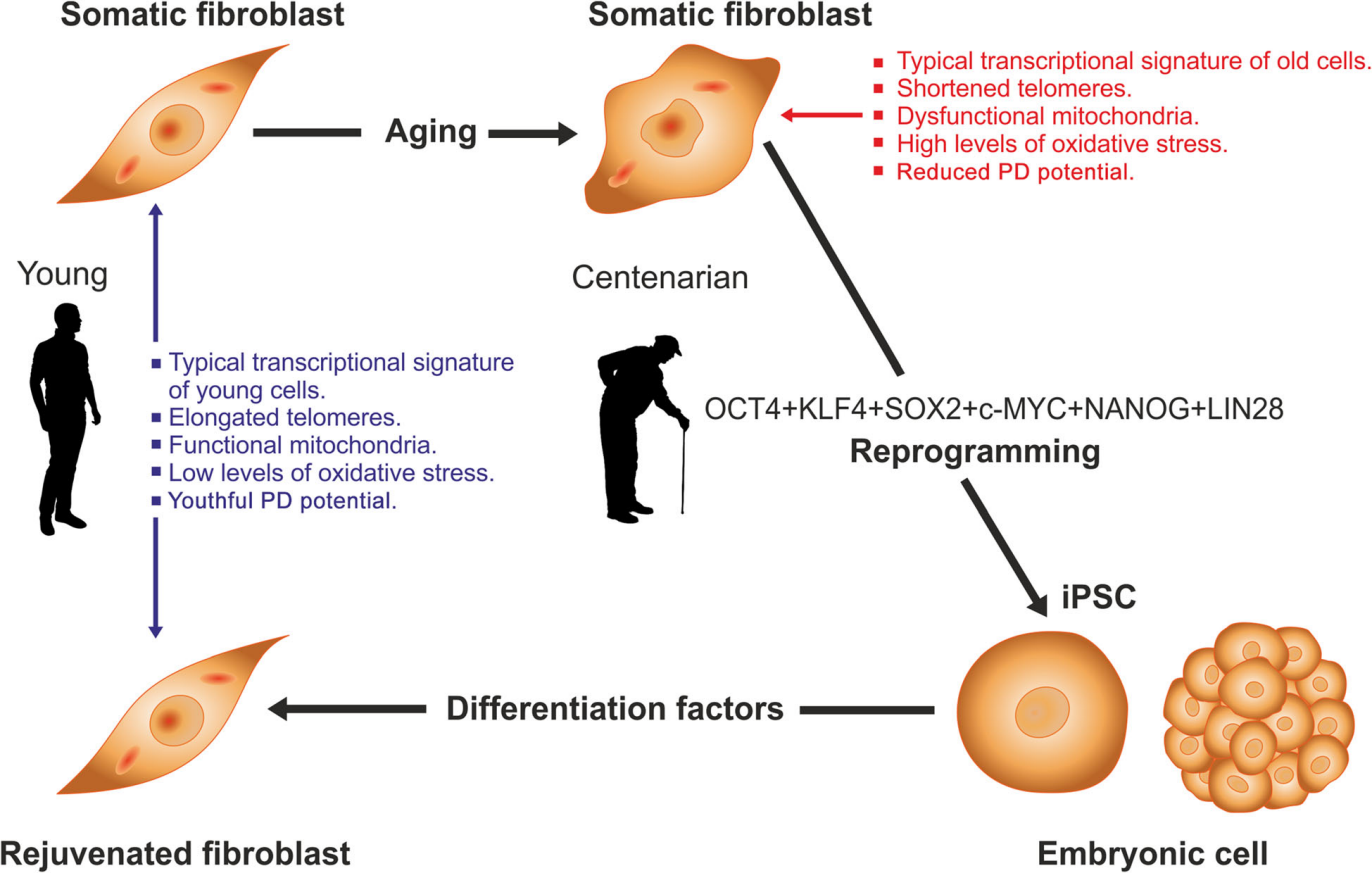
Rejuvenation by cell reprogramming of fibroblasts from healthy centenarian individuals. In culture, fibroblasts from old individuals display a typical transcriptional signature, different from that of young counterparts as well as shortened telomeres, reduced population-doubling (PD) potential, dysfunctional mitochondria, and higher levels of oxidative stress. When cells were reprogrammed to iPSC with a six-factor cocktail, the above alterations were reversed to embryonic levels. Then, iPSCs were differentiated back to fibroblasts by culture in the presence of an appropriate set of differentiation factors. In the resulting cells, all of the above variables had levels typical of fibroblasts taken from young individuals, see [4] for further details

It was known that cells from old individuals display a typical transcriptional signature, different from that of young counterparts [5]. It was also known that fibroblasts from old donors have shortened telomeres [6] as well as dysfunctional mitochondria and higher levels of oxidative stress [7]. The French group first explored the effect of cell reprogramming on the above features. In order to efficiently reprogram fibroblasts from healthy centenarians and very old donors, the authors added the pluripotency genes NANOG and LIN28 to the OSKM reprogramming cocktail. This six-factor combination efficiently reprogrammed fibroblasts from very old donors into typical induced pluripotent stem cells (iPSCs) (Fig. 1). These blastocyst-like cells showed a higher population-doubling (PD) potential than the cells of origin as well as elongated telomeres and a youthful mitochondrial metabolism (estimated by measuring mitochondrial transmembrane potential and clustering transcriptome subsets involved in mitochondrial metabolism). Using an appropriate differentiation cocktail, the iPSCs were differentiated back to fibroblasts, whose transcriptional profile, mitochondrial metabolism, oxidative stress levels, telomere length, and PD potential were indistinguishable from those of fibroblasts from young counterparts. Taken together, the data revealed that the cells had been rejuvenated.

After Lapasset et al.’s paper, a core of independent studies followed which confirmed the initial findings. Thus, reprogramming of skin fibroblasts from aged humans to iPSC, followed by differentiation to induced neurons (iNs), was shown to rejuvenate their transcriptome profile and nucleocytoplasmic compartmentalization (NCC) to that of wild-type fibroblasts from young donors [5]. When iNs were generated by transdifferentiation, a procedure that bypasses the pluripotency stage, the resulting neurons retained the transcriptome signature of old fibroblasts and showed a disrupted NCC as old fibroblasts do, which led to the conclusion that transient dedifferentiation to the iPSC stage is necessary to rejuvenate cells [5]. It is now known that induced neurons generated by conventional reprogramming and pluripotency factor-mediated direct reprogramming (PDR) are rejuvenated whereas induced neurons generated by transdifferentiation (also known as lineage reprogramming (LR)) are not [8].

In an interesting study with skin fibroblasts from healthy aged volunteers, it was observed that the lower oxygen consumption typically observed in the mitochondria of cells from old individuals was restored to youthful levels after the old cells were dedifferentiated to iPSCs and subsequently differentiated back to rejuvenated fibroblasts [9].

In 2013, Nishimura et al. [10] reprogrammed clonally expanded antigen-specific CD8+ T cells from an HIV-1-infected patient to pluripotency. The T cell-derived iPSCs were then redifferentiated into CD8+ T cells that had a high proliferative capacity and elongated telomeres.

Recently, rejuvenation of dysfunctional hematopoietic stem cells (HSCs) from old mice was achieved by reprogramming them to the iPSC stage followed by differentiating them back to HSCs. It was found that the rejuvenated HSCs from old mice had the same functional performance concerning the production of different immune and erythroid cell lineages (peripheral B-, T-, and granulocyte/myeloid cells, as well as bone marrow erythroid progenitors) than HSCs from normal young mice [11].

Another study reported that overexpression of the pluripotency factor NANOG in progeroid or senescent myogenic progenitors reversed cellular aging and fully restored their ability to generate contractile force. The effect was mediated by the reactivation of the ROCK and TGF-β pathways [12]. For a more detailed description of references [5–11], see [13].

**Fig. 2:**
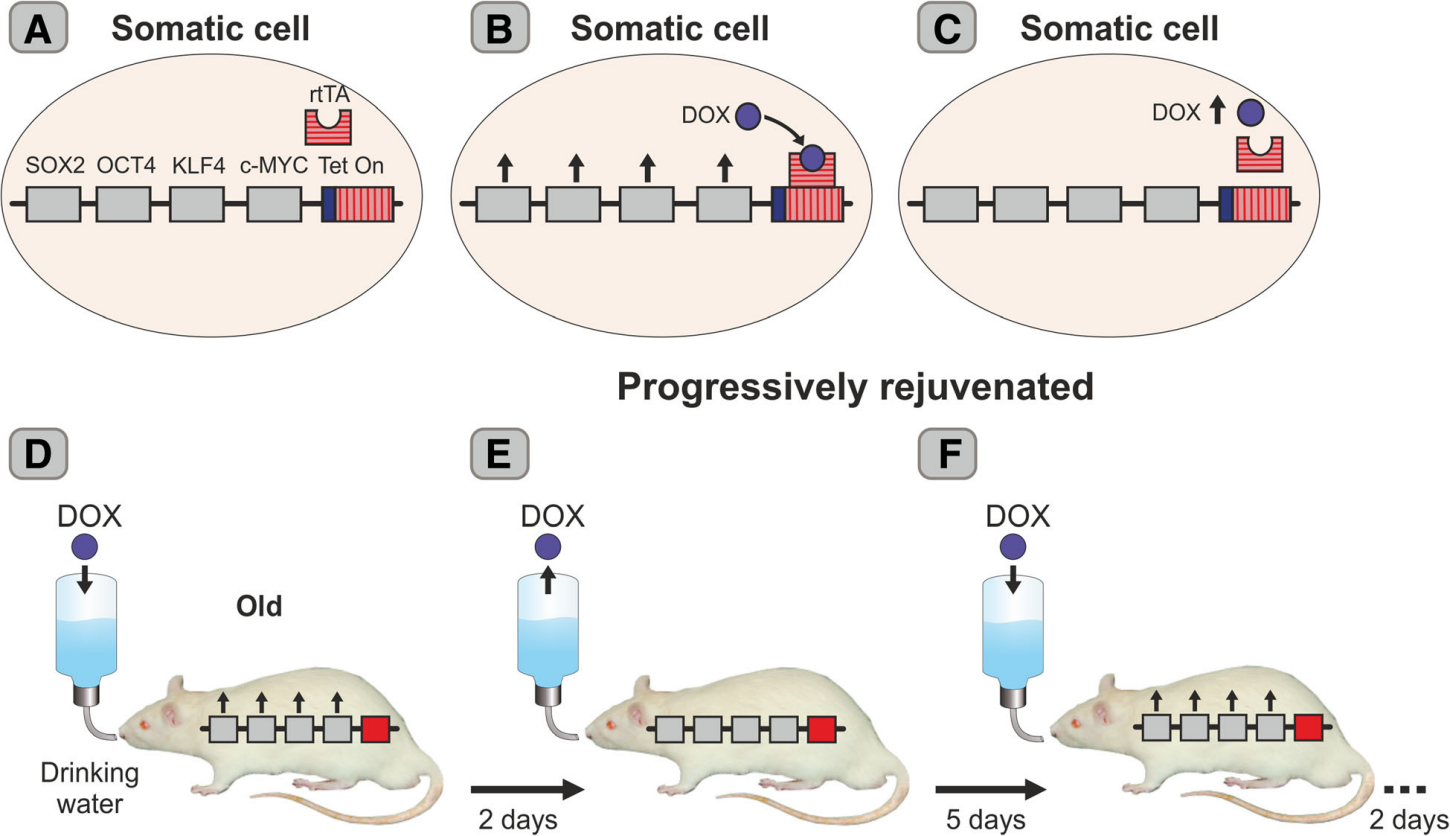
Rejuvenation of transgenic progeric mice by cyclic partial cell reprogramming. A polycistronic cassette (4F system) harboring the four Yamanaka genes under the control of a tetracycline-regulatable (Tet On) promoter (**a**) was transferred to one-cell embryos of C57bl/6 wild-type mice in order to generate transgenic mice harboring the 4F system (4F mice) that were subsequently backcrossed with transgenic progeric mice (LAKI mice). This way, progeric LAKI-4F mice were generated. The antibiotic doxycycline (DOX) binds to the regulatory rtTA protein which then gains affinity for the Tet On promoter and binds to it turning on the Yamanaka genes (**b**). When DOX is removed from the medium, the rtTA protein dissociates from the promoter and the transgenes become silent again (**c**). When DOX was added to the drinking water of 2-month-old progeric mice, it turned on the Yamanaka genes and partial cell reprogramming began (**d**). Two days later, DOX was removed and the Yamanaka genes silenced (**e**). After a 5-day resting period, DOX was added again for 2 days (**f**), then removed for 5 days and so on. This cyclic partial reprogramming process rejuvenated some tissues and organs of the mice which survived 50% longer than the original progeric mice, see [16] for further details

Until late 2016, it was believed that although cells taken from old individuals could be fully rejuvenated, rejuvenation in vivo was not possible as a continuous expression of the Yamanaka genes in animals had been shown to cause multiple teratomas [14, 15]. However, in December 2016, it was reported for the first time that cells and organs can be rejuvenated *in vivo* [16]. Since it was a quantum leap in rejuvenation technology, the study will be described in some detail. The authors used transgenic progeric mice (LAKI mice) harboring a mutated form of the human gene *Lmna* which causes the accumulation of a truncated form of the nuclear membrane protein Lamin A (progerin) present in progeric patients. They also used transgenic wild-type mice (4F mice) harboring two constructs. On chromosome 11, there was a construct that expresses the OSKM genes which are driven by a tetracycline-responsive (TRE) promoter, and on chromosome 6, an expression cassette was cloned which expresses the rtTA regulatory protein. When the rtTA protein binds the antibiotic doxycycline (DOX), a conformational allosteric change takes place so that rtTA gains affinity for the TRE promoter turning on the Yamanaka gene tandem [16]; (Fig. 2a–c). After repeated backcrossings, progeric transgenic mice expressing the 4F system (LAKI-4F mice) were generated [16]. Since the LAKI-4F mice age prematurely, by 2 months of age, they are already senile. Thus, at age 2 months, LAKI-4F mice began to be submitted to a rejuvenation strategy based on the addition of DOX to the drinking water of experimental mice in order to turn on the Yamanaka genes; 2 days later, the antibiotic was removed from the drinking water so that the transgenes were silenced. Mice rested for 5 days after which DOX was added again for 2 days then removed for 5 days and so on, thus implementing a cyclic partial reprogramming strategy (Fig. 2d–f). After 6 weeks of partial reprogramming cycles, the experimenters could observe some improvements in the external appearance of experimental mice, including a reduction in spine curvature as compared with untreated counterparts (controls). A subgroup of the experimental and control mice was sacrificed and some of their tissues and organs analyzed (skin, kidneys, stomach, and spleen). Controls showed a variety of alterations at an anatomical and histological level in the above organs whereas some of these aging signs disappeared or were attenuated in the experimental mice. Some aging signs remained unchanged by the treatment. Furthermore, although the experimental animals kept aging, they showed a 50% increase in mean survival time as compared with wild-type progeric controls. If the treatment was interrupted, the aging signs came back.

An important implication of the study by Ocampo et al. is that cyclic partial reprogramming causes some epigenetic aging marks to be erased, but spares the differentiation marks, which in turn suggest that both types of epigenetic mark are not necessarily the same.

## Theories of aging: cumulative DNA damage versus epigenetic theories

### Cumulative DNA damage

**Fig. 3:**
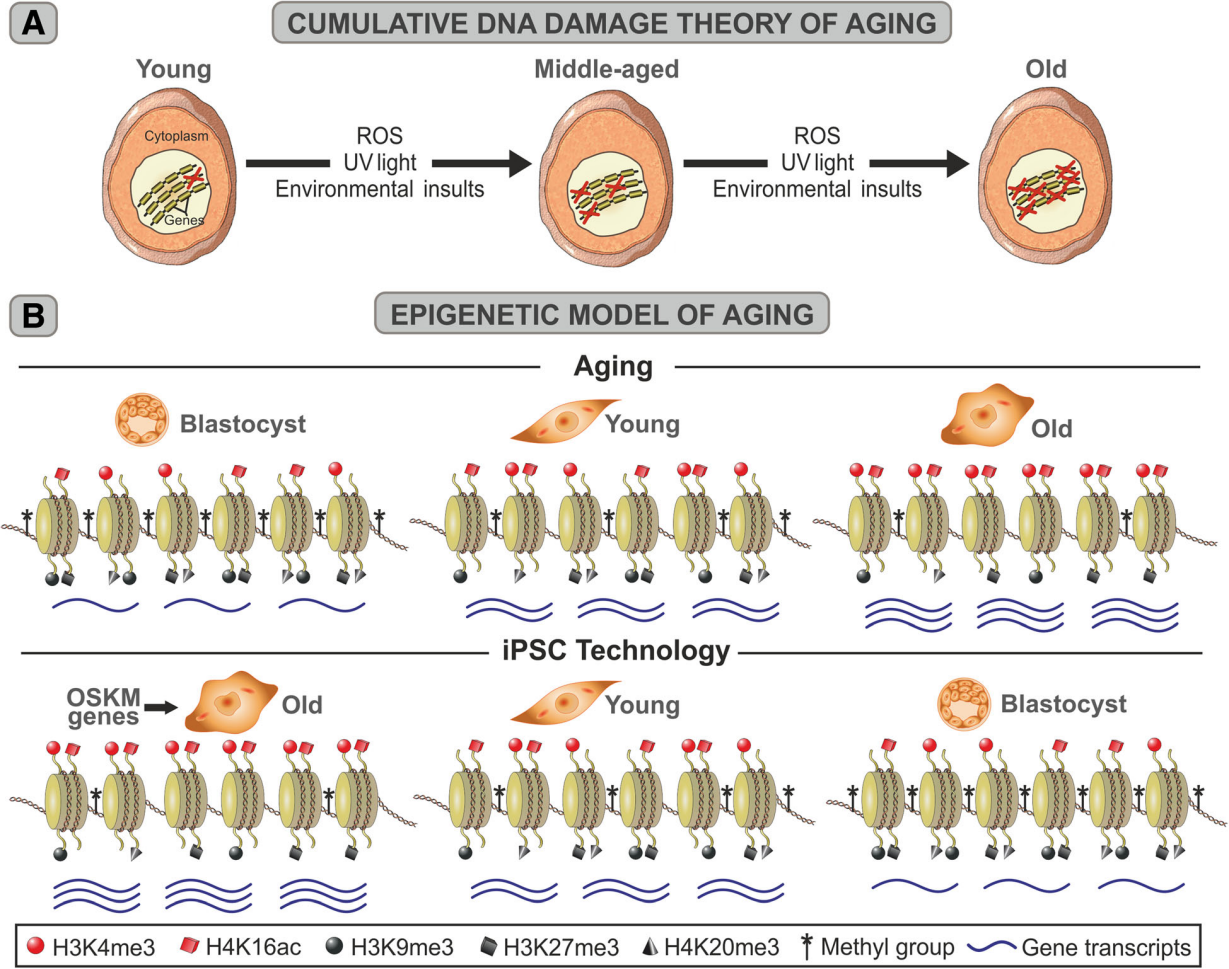
Diagrammatic representation of the cumulative DNA damage theory and the epigenetic model of aging. **a** Progressive age-related DNA damage that takes place in the genome of cells with age due to environmental insults. ROS, reactive oxygen species. **b** The upper diagram represents some of the progressive changes in histones H3 and H4 methylation and acetylation during normal aging. Changes in DNA methylation are represented by stemmed asterisks on DNA. The lower diagram represents the chronologic changes that may occur on the same epigenetic marks during OSKM gene-induced rejuvenation/dedifferentiation. Red symbols represent chromatin activating marks whereas black symbols correspond to chromatin-repressor marks. Blue wavy lines represent the gene transcripts

For several decades now, the theory of cumulative DNA damage has remained widely accepted by mainstream gerontologists. Basically, it proposes that aging is the consequence of the progressive accumulation of oxidative damage to cell macromolecules, particularly DNA [17, 18]. This phenomenon is thought to be especially relevant in mitochondria where respiration is associated with a continuous generation of reactive oxygen species as byproducts of O_2_ reduction (Fig. 3a) [19, 20]. According to this model, the process is essentially irreversible and can only be slowed down by appropriate interventions like calorie restriction [21]. Indeed, the idea that a progressive age-related deterioration of such an important molecule as DNA should play a central role in the aging process sounds sensible. However, experimental data from different sources do not support this model. For instance, the naked mole rat, a small rodent whose average lifespan (28.3 years) is nearly eight times longer than it is in laboratory rodents, has been shown to possess high levels of oxidative damage in its cells even when animals are young [22, 23]. It is likely that under extreme environmental conditions such as a highly ionizing atmosphere, radioactive zones or high UV light intensities, DNA damage may become a longevity-determining factor. This will also be the case under normal environmental conditions in pathologies associated with DNA repair defects, like Xeroderma pigmentosum. However, wherever normal marine or terrestrial earth conditions prevail, cumulative DNA damage does not seem to play a relevant role in aging.

### Epigenetic model

The growing evidence that reprogramming of somatic cells from aged individuals rejuvenates them to their embryonic stage is giving rise to the idea that the epigenome is the central driver of aging, at least in metazoans, and that the process is reversible [24].

Studies in model organisms like yeasts, worms, and flies have shown that aging is associated with progressive changes in chromatin regulation. In young cells, the genome is in a relatively high level of repression which is in part achieved by DNA methylation, and there are relatively high levels of histone H3 trimethylated at lysine 9 (H3K9me3) and at lysine 27 (H3K27me3) and histone H4 trimethylated at lysine 20 (H4K20me3), all of which are associated with transcriptionally repressed chromatin, as well as relatively low levels of histone H3 trimethylated at lysine 4 (H3K4me3) and histone H4 acetylated at lysine 16 (H4K16ac), both of which are associated with active chromatin [25]. Aging seems to be associated with a progressive derepression of the transcriptional activity of chromatin, which is effected in part by a reduction in DNA methylation; a decrease of epigenetic repressor marks like H3K9me3, H3K27me3, and H4K20me3 as well as an increase in the levels of activation marks like H3K4me3 and H4K16ac (Fig. 3b). Cell reprogramming technology shows that this process is reversible (Fig. 3b). The epigenetic model of aging provides an elegant explanation for a number of age-related processes difficult to explain by conventional theories of aging (see below).

### The epigenetic clock theory

An important advance relevant to the epigenetic model of aging is the relatively recent discovery that the level of age-related methylation of a set of cytidine-guanosine dinucleotides (CpG) located at precise positions throughout the genome constitutes a highly reliable biomarker of aging. A mathematical algorithm, the multi-tissue age predictor also known as the epigenetic clock, devised by Stephen Horvath [26], uses the age-dependent methylation state (beta value) of 353 CpGs located at precise positions through the human genome and generates a number, expressed in years, that represents epigenetic age. In humans, the epigenetic age generated by this predictor shows a correlation of 0.96 to chronological age and an error margin of 3.6 years [27]. This is an unprecedented accuracy for a biomarker of age, far superior to all biomarkers of age so far documented. Remarkably, the epigenetic clock predicts biological age with comparable high accuracy when applied to DNA taken from the whole blood, peripheral blood mononuclear cells, occipital cortex, buccal epithelium, colon, adipose, liver, lung, saliva, and uterine cervix [27]. The rate of change in DNA methylation at age-dependent CpGs represents the ticking rate of the epigenetic clock. The rate is very high in humans from birth to 1 year of age; from 1 to 20 years of age, it progressively decelerates; and from age 20 onwards, it changes to a much slower rate [27]. It can also be said that the ticking rate of the epigenetic clock represents the changing rate of DNA methylation heterogeneity among cells in tissues. Now, it seems clear that lifespan is associated not to the number of total tickings of the epigenetic clock but to the rate of ticking after 20 years of age when a human has achieved maturity. There is compelling evidence that genetics is the primary controller of the rate of epigenetic aging. For instance, it is known that the rate of epigenetic aging is slower in supercentenarians and their descendants [28]. It should be pointed out that the epigenetic clock is an accurate predictor of chronological age mainly in a context of normal aging; under pathological circumstances, the epigenetic age displayed by the clock represents biological rather than chronological age. For instance, pathologies like Huntington’s and Parkinson’s diseases are associated with accelerated epigenetic aging [29, 30]. Consistent with the epigenetic model of aging, when 30-year-old human [26] and 2-month-old mouse [31] somatic cells are reprogrammed to iPSCs by the Yamanaka factors, their epigenetic age reverts to zero years or months, respectively. The epigenetic clock theory of aging hypothesizes that biological aging is an unintended consequence of both developmental and epigenetic maintenance programs for which the molecular footprints give rise to DNA methylation (DNAm) age estimators [32]. Indeed, the epigenetic clock theory and the epigenetic model of aging go hand in hand and support each other.

## The theories at work

**Fig. 4:**
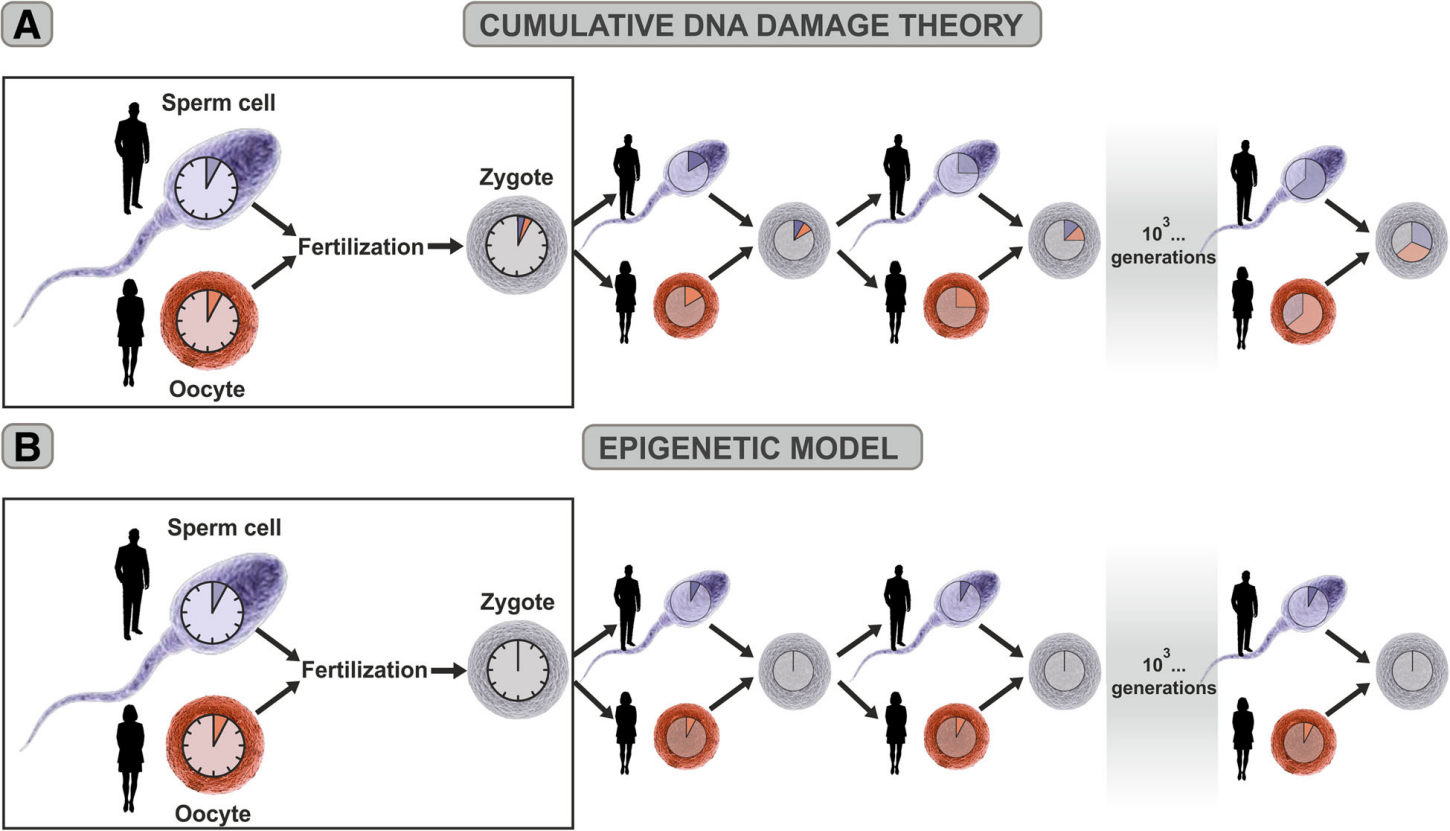
The two theories at work. **a** According to the cumulative DNA damage theory, when a hypothetical 25-year-old human couple conceives a new individual, the zygote they conceived inherits the DNA damage of the parental germ cells. (Left enlarged panel) According to the theory, after each generation, DNA damage in the successive zygotes would be accumulated through inherited damage, causing species viability to decline progressively over the centuries, eventually driving them to extinction. (Main diagram) According to the epigenetic model, at the time of fertilization, all the epigenetic marks of parental aging are erased from the zygote’s genome by the reprogramming factors present in the cytoplasm, thus resetting its aging clock back to zero (**b**, left enlarged panel). Consequently, in each generation, the epigenetic clock of zygotes will restart from zero, thus allowing that complex animal species flourish and diversify over time (**b**, main diagram)

The conception of a new individual involves the genesis of a completely young organism which does not inherit any aging trait from its parents, after conception life starts anew. According to the cumulative DNA damage theory, when a hypothetical human couple, 25 years of age each member, conceive a new individual, the DNA damage accumulated in the genome of their germ cells during 25 years is probably minimal but higher than zero. Therefore, the new zygote should inherit that minimal damage (Fig. 4a, enlarged inset), and as a consequence, it would not be fully young. In contrast, according to the epigenetic model, the zygote conceived by the same couple would have its epigenetic clock reset to zero by the reprogramming factors present in the cytoplasm of that cell (Fig. 4b, enlarged inset). This hypothesis is based on the fact that when human or mouse adult somatic cells are reprogrammed to iPSCs, their epigenetic age is reset virtually to zero [26, 31].

While every individual of a metazoan species ages, complex animal life has remained viable over hundreds of million years. According to the cumulative DNA damage theory, germ cells are likely to sustain some accumulation of DNA damage in spite of the fact that DNA is highly protected in this type of cells. Since complex animal life emerged during the era of life evolution known as the Cambrian explosion, some 550 million years ago [33], the enormous number of generations of germ cells that took place over the successive millennia during which many complex animal species evolved should have accumulated increasingly large amounts of DNA damage that would be inherited by the zygotes they conceive (Fig. 4a, main panel). Such accumulating DNA damage should cause complex animal species to progressively weaken and eventually become extinct, which obviously is not the case.

According to the epigenetic model, at the time of fertilization, all the epigenetic marks of parental aging are erased from the zygote’s genome, resetting its aging clock back to zero. Therefore, in each generation, the epigenetic clock of zygotes will restart from zero (Fig. 4b, main panel), thus allowing that complex animal species flourish and diversify over the millennia.

## Therapeutic potential of cyclic partial reprogramming

Besides showing that the OSKM genes are able to partially rejuvenate cells and organs in mice, the same study described above, Ocampo et al. [16], used 12-month-old, transgenic nonprogeroid mice. Cyclic partial reprogramming enhanced the otherwise poor regenerative capacity of their pancreas and skeletal muscle and made these tissues more resilient to a subsequent insult.

Specifically, cyclic transient induction of OSKM triggered proliferation of beta cells in the pancreas and satellite cells in the skeletal muscle, which are critical for the maintenance of tissue homeostasis, but whose numbers typically decrease with age. Therefore, the benefits of cyclic partial reprogramming may go beyond rejuvenation of old animals; it could also constitute an effective regenerative treatment [34].

Concerning in vivo rejuvenation, the main challenges that partial reprogramming in vivo presents are the need to employ an approach that does not use transgenic animals and that does not require indefinitely long application of the method to prevent the rapid return of the aging marks after termination of the treatment. Once such hurdles are overcome, clinical treatment will become potentially feasible. In such case, the first, more amenable, clinical approach would be a preventive one in which adult individuals carrying risk factors for late-life diseases are submitted to a partial cell reprogramming approach not necessarily to rejuvenate them but to stop or at least significantly slow down their aging rate. In a more advanced stage of the partial reprogramming technology, progressive rejuvenation of old individuals could be attempted. At the increasing pace of technology advancement, the above therapeutic aims may become available routines in the not-too-distant future.

The possibility that human rejuvenation strategies may become routine treatments raises some ethical issues, a relevant one being a potential overpopulation of the planet with the consequential impact on the availability of food, energy, and other commodities. In any case, these problems lie in the relatively distant future, and it is to be hoped that more advanced societies will establish regulatory legislation aimed at preventing a demographic explosion.

## Concluding remarks

The discovery of animal cloning and subsequent development of cell reprogramming technology were quantum leaps as they led to the achievement of rejuvenation by cell reprogramming which in turn constitutes a paradigm shift in gerontology. A major consequence of these discoveries is the emerging view that aging is an epigenetic process and that somewhere in the genome of somatic cells, there is a viable genetic program that can be set in motion by a small number of master genes. The purpose of this putative program seems to be the reprogramming of somatic cells into blastocyst-like, pluripotent embryonic stem cells. Although at present we know virtually nothing about the location of this hypothetic rejuvenation program, let alone its structure and mechanism of action, we do know how to turn on the program even in somatic cells from very old individuals. We have discovered a powerful tool designed by nature and perfected during millions of years of evolution and have learned how to use it to rejuvenate cells and to a certain extent, animals. The goal of understanding the molecular mechanisms involved in pluripotency factor-mediated rejuvenation is not nearly as imperative as the objective of learning how to implement cell reprogramming technology in nontransgenic old animals and humans in order to erase epigenetic marks of aging without removing epigenetic marks of cell identity. The rate of progress in biotechnology is increasingly fast, approaching an exponential curve. It is therefore reasonable to expect that clinical approaches to stop aging and even rejuvenate aged humans will be developed within the next two or three decades.

It has been stated that “any sufficiently advanced technology is indistinguishable from magic” [35]. Now, it seems that the magic of human technology is making rejuvenation possible.

## Abbreviations

CpG: Cytidine-guanosine dinucleotide
DOX: Doxycycline
H3K27me3: Histone 3 trimethylated at lysine 27
H3K4me3: Histone H3 trimethylated at lysine 4
H3K9me3: Histone H3 trimethylated at lysine 9
H4K16ac: Histone H4 acetylated at lysine 16
H4K20me3: Histone H4 trimethylated at lysine 20
HSC: Hematopoietic stem cell
iN: Induced neuron
iPSC: Induced pluripotent stem cell
NCC: Nucleocytoplasmic compartmentalization
OSKM genes: *oct4*, *sox2*, *klf4*, and *c-myc*
